# Supplementary material for: Catfish Egg Lectin Enhances the Cytotoxicity of Sunitinib on Gb3-Expressing Renal Cancer Cells
Source: Biomedicines. 2023 Aug 21;11(8):2317. doi: 10.3390/biomedicines11082317 (PMC10452590; doi:10.3390/biomedicines11082317)
Supplement: Supplementary file 1 [file biomedicines-11-02317-s001.zip › biomedicines-2556013-supplementary.pdf]

Supplementary Materials

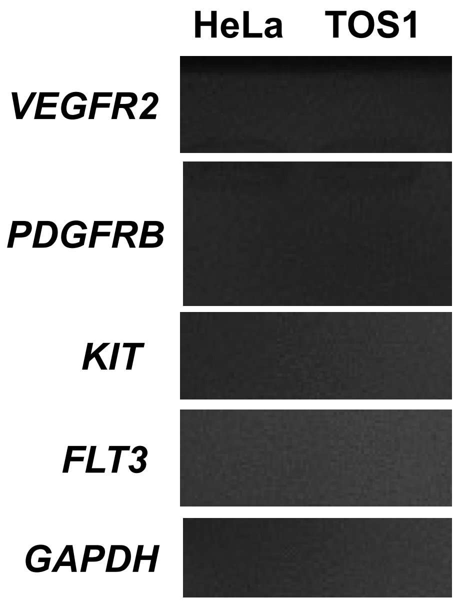

Supplementary Figure S1. Expression of tyrosine kinase receptor genes on HeLa and TOS1 cells. Total RNA extracted from HeLa and TOS1 cells (5×10^4) was analyzed by RT-qPCR using specific primers for *VEGFR2*, *PDGFRB*, *KIT*, *FLT3*, and *GAPDH*. PCR products were analyzed on 2% agarose gels.

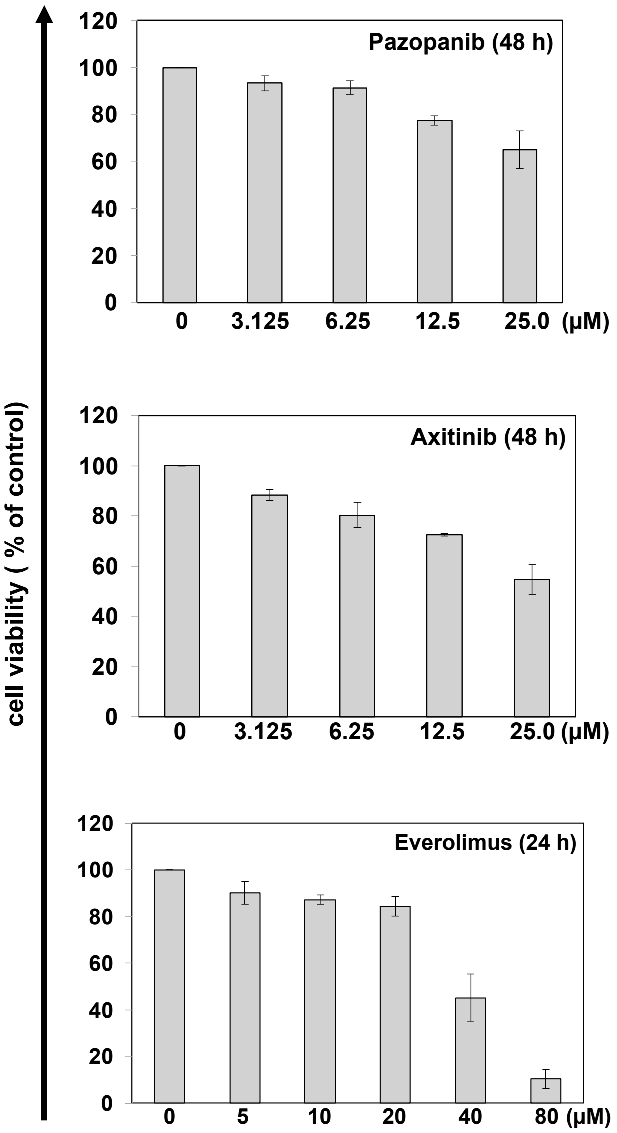

Supplementary Figure S2. Cytotoxic effects of pazopanib, axitinib, and everolimus in TOS1 cells. TOS1 cells were treated with pazopanib (0, 3.125, 6.25, 12.5, and 25 μM), axitinib (0, 3.125, 6.25, 12.5 and 25 μM), or everolimus (0, 5, 10, 20, 40 and 80 μM) at 37 °C for 24 or 48 h. Cell viability was measured using the CCK-8 assay. Values represent means ± standard errors (SEs) of three independent experiments.

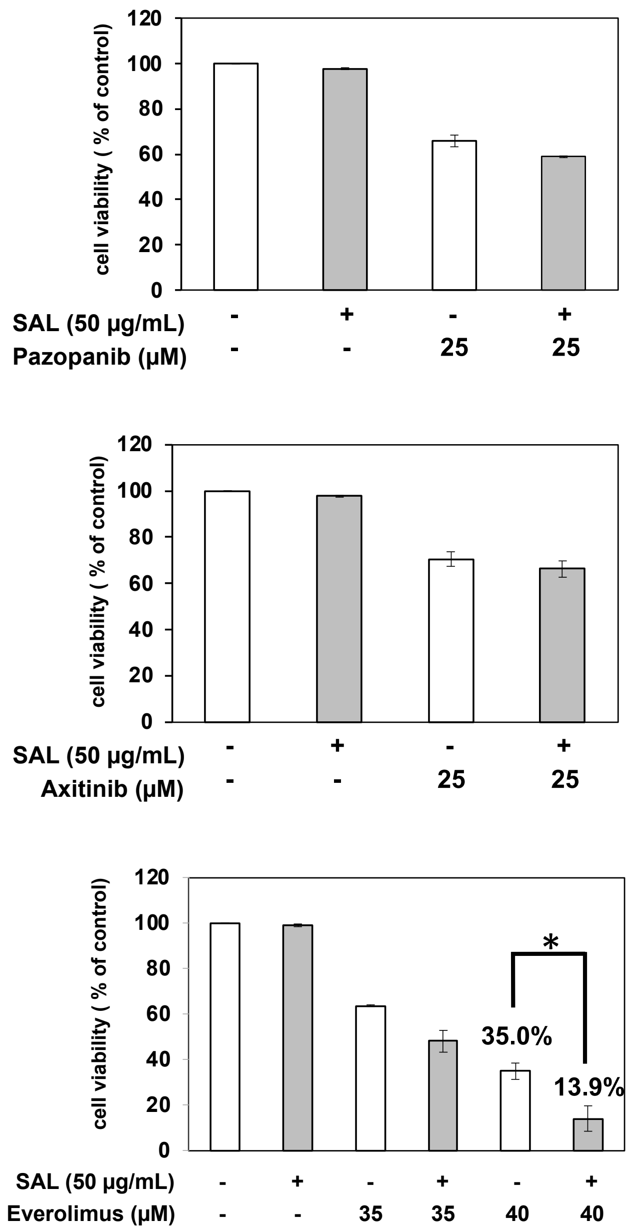

Supplementary Figure S3. Cytotoxic effects of pazopanib, axitinib, and everolimus in SAL-pretreated TOS1 cells. TOS1 cells were pretreated with SAL (50 µg/mL) at 37 °C for 24 h. The SAL-pretreated cells TOS-1 cells were treated with pazopanib (0 and 25 µM), axitinib (0 and 25 µM), or everolimus (0, 35, and 40 µM) at 37 °C for 24 or 48 h. Cell viability was measured by the CCK-8 assay. Values represent means ± SEs of three independent experiments. $*p < 0.05$ *versus* non-treated control cells.
